# Convergence and divergence of B cell responses in two HIV-1 Env immunizations in Rhesus macaques

**DOI:** 10.1038/s43856-025-00899-3

**Published:** 2025-05-15

**Authors:** Jenna M. DeLuca, Maria Blasi, Taylor J. McGee, Shalini Jha, Xiaoying Shen, Shuqin Gu, Justin Pollara, Melissa Kerkau, Mansi Purwar, Diane G. Carnathan, Donatella Negri, Andrea Cara, Kurt Wollenberg, Kevin Wiehe, Kevin O. Saunders, Shan Lu, Guido Silvestri, David B. Weiner, Mary E. Klotman, Guido Ferrari, M. Anthony Moody, Mattia Bonsignori

**Affiliations:** 1 Translational Immunobiology Unit, Laboratory of Infectious Diseases, National Institute of Allergy and Infectious Diseases, National Institutes of Health, Bethesda, MD USA; 2 Duke Human Vaccine Institute, Duke University, Durham, NC USA; 3 Department of Medicine, Duke University, Durham, NC USA; 4 Department of Surgery, Duke University, Durham, NC USA; 5 Wistar Institute, Philadelphia, PA USA; 6 Emory National Primate Research Center and Emory Vaccine Center, Emory University School of Medicine, Atlanta, GA USA; 7 Department of Infectious Diseases, Istituto Superiore di Sanità, Rome, Italy; 8 National Center for Global Health, Istituto Superiore di Sanità, Rome, Italy; 9 Bioinformatics & Computational Biosciences Branch, National Institute of Allergy and Infectious Diseases, National Institutes of Health, Bethesda, MD USA; 10 University of Massachusetts Medical School, Worcester, MA USA; 11 Department of Pediatrics, Duke University, Durham, NC USA

**Keywords:** Protein vaccines, Immunological memory

## Abstract

**Background:**

Sequential multivalent immunizations are used to counter diversity in rapidly mutating viruses. Here, we evaluated the effect of HIV-1 immunogen formats on the binding profile of memory B-cells elicited in two independent Rhesus macaque trials.

**Methods:**

In one trial, female Rhesus macaques were immunized with a multiclade HIV-1 gp120 envelope glycoprotein (Env) cocktail and bled two weeks post final immunization. In another trial, male and female Rhesus macaques were sequentially immunized with clonally-related Env glycoproteins: Four immunogens were administered as non-stabilized gp140 Envs and the fifth as a specially stabilized gp140 Env trimer (SOSIP); animals were bled before and after SOSIP immunization. Immunogen-binding peripheral memory B-cells were sorted and cultured at limiting dilution. Culture supernatants were assessed by ELISA for binding to individual immunogens.

**Results:**

In the first trial, 81% (591/734) of B-cells cross-react with multiple Envs and most bind to all immunogens. In the second trial, 81% (331/410) of B-cells isolated before SOSIP administration react with all non-stabilized gp140 Env immunogens and 27% also cross-react with the yet-to-be-administered SOSIP-stabilized Env. However, after SOSIP administration, SOSIP-stabilized trimer-reactive B-cells increase to 86% (219/256) but most (82%) do not cross-react with the preceding immunogens.

**Conclusions:**

Multiclade and sequential regimens before SOSIP-stabilized Env immunization elicited B-cells that converge on shared epitopes. A change in immunogen format results in a divergent B-cell response that vastly fails to engage prior responses. Critically, B-cell priming with non-stabilized Env cannot modify the effect of the epitope immunodominance hierarchy in a SOSIP trimer. These results suggest that a change in immunogen format may cause off-target B-cell engagement, but also that B-cell repriming is possible despite pre-existing immunity.

## Abstract (web summary)

DeLuca et al. evaluate the effect of HIV-1 immunogen formats on the binding profile of memory B cells from two independent non-human primate trials. These results show how immunogens with differing immunodominance hierarchies can induce selective B cell response in the context of sequential immunizations.

## Plain Language Summary

An effective vaccine against the human immunodeficiency virus-1 (HIV-1) will likely require a complex design. We studied how immune memory could be elicited and recalled by testing two vaccine strategies in rhesus macaques, animals similar to humans in immune response. In the first study, macaques received a mix of HIV-1 components, called proteins, that led to most of them being recognised by the host immune cells and generating immune memory for future exposure. In the second study, macaques were given a series of related HIV-1 proteins, ending with a specific protein called SOSIP. This led to SOSIP being recognised by a limited immune memory generated against the proteins given earlier. This highlights the importance of immune memory for repeated doses of different HIV-1 vaccines.

## Introduction

Successful vaccines against highly mutating viruses, such as influenza virus and SARS-CoV-2, rely on sequential immunizations with mono- or multivalent formulations to counter viral diversity and broaden the spectrum of primed B cells at the time of exposure^1^.

For HIV-1, multivalent, multiclade vaccination schemes - a method historically used to counter viral diversity - and sequential immunization strategies with clonally related HIV-1 envelope glycoproteins (Env) have been pursued^2^. The ability of B cells to bind to a single *versus* multiple immunogens (“cross-reactivity” for brevity from here onward) in the context of multivalent immunization regimens has been examined for many vaccine targets including HIV-1 Env (e.g. refs. ^3,4^), influenza hemagglutinin^5,6^, and even for antigens with few shared epitopes (e.g., influenza hemagglutinin and anthrax protective antigen)^7^. However, while the ability to induce cross-reactive B cells has been demonstrated, it is not yet fully known what rules dictate the recall and maturation of earlier responses versus the elicitation of de novo responses to related antigens for novel immunogens.

To assess this, we analyzed the cross-reactivity of B cells isolated from non-human primates (NHPs) immunized with either a multiclade vaccine formulation or sequential immunizations with clonally related non-stabilized gp140 Envs followed by a specially stabilized gp140 Env trimer (SOSIP). The Rhesus macaque and human immune systems share significant similarities. Therefore, Rhesus macaques are widely used in HIV research as a non-human primate model to assess in a controlled environment the immune responses elicited by vaccine candidates without experimenting directly on humans.

Herein, we demonstrate that both multivalent and sequential HIV-1 Env immunogen regimens can elicit cross-reactive B cell responses in rhesus macaques. We further demonstrate that for the sequential regimen, a change in immunogen format to a SOSIP-stabilized gp140 Env trimer engaged a novel population of B cells, despite the presence of SOSIP-stabilized gp140 Env trimer cross-reactive B cells prior to the boost.

## Methods

### NHP multivalent immunization with a multiclade Env formulation

The four NHPs included in this study (RNs14, RVv15, RPt15 and RSf15) were selected from an NHP trial designed to evaluate the effect of adjuvants in a DNA prime/polyvalent protein boost immunization strategy. Briefly, 40 NHPs were divided into 4 groups. One control group received mock immunizations (*n* = 10). All other groups were primed three times (weeks 0, 4 and 8) with polyvalent HIV-1 gp120 envelope DNA (2 mg/construct) either without adjuvant (Group A), with pIL-12 (Group B) or with pMec/CCL28 (Group C). RVv15, RNs14 and RPt15 were included in Group A and RSf15 was from Group B. The polyvalent DNA preparation included the following DNAs: A.Q23.17, A.Q259d2.17, B.WITO4160.33, B.CAAN5342.A, B.THRO4156.18, C.Du172.17, C.ZM214M.PL15, and clade A, B and C consensuses. The first two immunizations were delivered i.d. with electroporation and the third immunization was delivered i.m. with electroporation. All groups were boosted twice i.m. (weeks 12 and 16) with 0.2 mg of a tetravalent gp120 Env protein cocktail (50 µg/protein) comprising clades A (92UG037), B (JR-FL), C (93MW965) and CRF AE (gp120 AE consensus) HIV-1 strains in Monophosphoryl Lipid A (synthetic) PHAD-(MPLA) adjuvant (0.4 mg). Peripheral blood mononuclear cells (PBMCs) were collected 14 days after the last immunization. All immunizations were delivered in the right thigh. NHPs were challenged intravaginally weekly up to 15 times with 200 TCID_50_ of heterologous clade C SHIV.CH505.375H.dCT strain. The rhesus macaques used in this study were housed at Emory University in accordance with the recommendations of the Association for Assessment and Accreditation of Laboratory Animal Care International Standards and with the recommendations in the Guide for the Care and Use of Laboratory Animals of the United States - National Institutes of Health. The study was approved by the Emory University Institutional Animal Use and Care Committee (PROTO201800112).

### Plasma viral load determination

Quantitative real-time reverse-transcriptase (RT)-PCR assay to determine SHIV.CH505.375H.dCT viral load was performed as previously described^8^. Briefly, we measured the number of viral RNA copy number in 140 µl of ACD plasma. The samples were manually extracted via QIAamp Viral RNA kit (Qiagen), diluted in 60 µl, and frozen at −80 °C until RNA quantitation was performed. Fifteen µl of purified plasma RNA was reverse-transcribed using the High Capacity cDNA Reverse Transcription Kit (Applied Biosystems). The gag primers used are listed as follows: forward 5’-GCAGAGGAGGAAATTACCCAGTAC-3’ and reverse 5’-CAATTTTACCCAGGCATTTAATGTT-3’. A PerkinElmer Applied Biosystems 7700 Sequence Detection System was used with the PCR profile: 95 °C for 10 min, followed by 40 cycles at 93 °C for 30 s, and 59.5 °C for 1 min. PCR product accumulation was monitored with the 7700 sequence detector and a probe to an internal conserved *gag* gene sequence: 5’ 6FAM-TGTCCACCTGCCATTAAGCCCGA-TAMRA-3’, where FAM and Tamra denote the reporter and quencher dyes. The resulting viral RNA copy number was determined utilizing a standard curve consisting of SIVmac239 RNA quantified by the SIV bDNA method (Bayer Diagnostics). All specimens were extracted and amplified in duplicate, with the mean result reported. With a 140 µl plasma input, the assay has a sensitivity of 60 RNA copies/ml of plasma.

### NHP sequential immunization with IDLV expressing CH505 Envs

The study design has been previously reported in detail^9^. Briefly, NHPs were immunized at 6-month intervals with non-stabilized gp140 Envs isolated from individual CH505, including the transmitted founder virus and sequentially evolved Envs isolated from weeks 53, 78 and 100 selected based on their binding profile to monoclonal antibodies of the CH103 broadly neutralizing antibody (bnAb) lineage at progressive maturation stages (CH505.T/F, CH505.w53, CH505.w78, CH505.w100, respectively). Two additional immunizations were performed using stabilized CH505.w136 SOSIP Env trimer. Immunogens were delivered either through IDLV alone (3 × 10^8^ TU i.m.) or in combination with the respective protein (100 µg s.c.) in GLE-SE adjuvant. Six weeks after the last immunization, NHPs were challenged intrarectally with the autologous SHIV.CH505.375H.dCT strain. PBMCs were collected 14 days after the last immunization with CH505.w78 gp140 (week 75) and CH505.w100 SOSIP (week 117). The rhesus macaques used in this study were housed at BIOQUAL, Inc. in accordance with the recommendations of the Association for Assessment and Accreditation of Laboratory Animal Care International Standards and with the recommendations in the Guide for the Care and Use of Laboratory Animals of the United States - National Institutes of Health. The study was approved by the BIOQUAL Institutional Animal Use and Care Committee (Study # 18-001).

### Serum neutralization assay

Serum was collected four weeks prior to immunization along with weeks 10 and 18 post-immunization. Env-pseudotyped virus neutralization assays were measured as a function of reductions in luciferase (Luc) reporter gene expression after a single round of infection in TZM-bl cells^10,11^. TZM-bl cells (also called JC57BL-13) were obtained from the NIH AIDS Research and Reference Reagent Program, as contributed by John Kappes and Xiaoyun Wu. Briefly, a pre-titrated dose of virus was incubated with serial dilutions of heat-inactivated (56 °C, 30 min) serum samples in duplicate for 1 h at 37 °C in 96-well flat-bottom culture plates, followed by addition of freshly trypsinized cells. One set of control wells received cells + virus (virus control) and another set received cells only (background control). After 48 h of incubation, cells were lysed and measured for luminescence using the Britelite Luminescence Reporter Gene Assay System (PerkinElmer Life Sciences). ID_50_ neutralization titers are the dilution (serum/plasma samples) at which relative luminescence units (RLU) were reduced by 50% compared to virus control wells after subtraction of background RLUs from cells-only controls. Plotted data are the highest serum neutralization values (peak ID_50_) against each virus throughout the timecourse for the four NHPs in this study.

### Memory B cell staining, sorting and culturing

We have previously described the memory B cell culture method adapted to induce proliferation and differentiation of Rhesus memory B cells into antibody-secreting cells^12,13^. Briefly, anticoagulated blood was obtained from NHPs at the timepoints specified. PBMC were isolated by density gradient centrifugation and stored cryopreserved in vapor phase of LN_2_ prior to thawing for analysis. Samples were thawed by incubating in a 37 °C water bath followed by washing with warmed media before being stained with CD20 FITC (BD Biosciences, catalog no. 347673, clone L27) or CD20 BV650 (BD Biosciences, catalog no. 740439, clone L27); CD3 PerCP-Cy5.5 (BD Biosciences, catalog no. 552852, clone SP34-2), IgD PE (Southern Biotech, catalog no. 2030-09, polyclonal); CD8 PE-Texas Red (Invitrogen, catalog no. MHCD0817, clone 3B5); IgM PE-Cy5 (BD Biosciences, catalog no. 551079, clone G20-127); CD16 PE-Cy7 (BD Biosciences, catalog no. 557744, clone 3G8); CD27 APC-Cy7 (BioLegend, catalog no. 302816, clone O323); CD14 BV570 (BioLegend, catalog no. 301832, clone M5E2); and Aqua Dead Cell Stain (Invitrogen, catalog no. L34957), as well as fluorochrome labeled Env immunogens pertinent to each study. All antibodies were titered in advance and used at optimal concentrations. Gp120 Env immunogens from the multiclade multivalent trial were directly biotinylated using NHS-PEG4-Biotin (ThermoFisher Scientific, catalog no. 21329) following manufacturers’ protocols. Non-stabilized and SOSIP-stabilized gp140 Env immunogens from the sequential immunization trial were expressed with an AviTag and biotinylated using site-specific BirA biotin-protein ligase reactions (Avidity, catalog no. BirA500) per the manufacturers’ instructions. Biotinylated proteins were tetramerized with streptavidin labeled with AF647 (Life Technologies/Invitrogen, catalog no. S21374), BV421 (Biolegend, catalog no. 405225), or VB515 (Miltenyi Biotec, catalog no. 130-108-993)^14^. For each study, all the pertinent Envs tagged with two fluorochromes were combined for staining and sorting as described^7^: specifically, PBMCs from the multivalent multiclade trial were stained with both AF647- and BV421-tagged A.92UG037, B.JR-FL, C.93MW965 and AE consensus gp120 Env, whereas PBMCs from the CH505 sequential trial were stained with both AF647- and VB515-tagged CH505.TF, CH505.wk53, CH505.wk78 and CH505.wk100 non-stabilized gp140 Envs, and SOSIP-stabilized CH505.wk136 gp140. Memory B cells were defined as CD3 negative, CD14 negative, CD16 negative, CD20 positive, CD27 all, IgD negative cells. Representative gating strategies for each of the two trials are shown in Supplementary Fig. S1. Viable memory B cells double-positive for AF647- and BV421-tagged Env immunogens, or AF647- and VB515-tagged gp140s and SOSIPs, were sorted (purity setting) using a BD FACSAria II (BD Biosciences, San Jose, CA) and cultured in bulk into wells containing 5000 MS40L feeder cells, RPMI-1640 supplemented with 15% FBS, 1 mM sodium pyruvate, 1% non-essential amino acids, 25 mM HEPES buffer, 2.5 μg mL^−1^ ODN2006 (Invivogen, Cat. no. TLRL-2006-5), 5 μM CHK2-inhibitor (Calbiochem, Cat. no. 220486-1MG), 100 ng mL^−1^ recombinant human interleukin (IL)−21 (Peprotech, Cat. no. 2001-21), 10 ng mL^−1^ recombinant Human BAFF (Peprotech, Cat. no. 310-13), 200 U mL^−1^ IL-2 (from the myeloma IL-2 producing cell line IL2-t6, kindly provided by Dr. Antonio Lanzavecchia, IRB, Bellinzona, Switzerland), and 100 μL supernatant of the Herpesvirus papio (HVP)-infected Baboon cell line S594 (NHP Reagent Resource). The concentration of each supplement was previously determined to achieve optimal in vitro stimulation. Following overnight incubation at 37 °C in 5% CO_2_, memory B cells were plated at limiting dilution in round bottom tissue culture 96-well plates containing 5000 non-irradiated MS40L feeder cells and cultured for 2 weeks. Culture medium was refreshed after 7 days and harvested 7 days later. Cells from each culture were archived in RNAlater (Qiagen) at −80 °C.

### ELISA culture supernatant screening

For the screening of the culture supernatants of the gp120 Env from the multivalent multiclade trial, high-binding 384-well plates were coated with 2 µg mL^−1^ gp120 Env proteins (15 µL/well) and blocked overnight with blocking buffer (PBS containing 4% [wt/vol] whey protein, 15% normal goat serum, 0.5% Tween-20 and 0.05% sodium azide) at 4 °C. For biotinylated non-stabilized and SOSIP-stabilized gp140 Env glycoproteins, high-binding 384-well plates were pre-coated with 2 µg mL^−1^ streptavidin (15 µL/well), incubated at RT for 2 h and blocked overnight at 4 °C. Biotinylated proteins (10 µL/well) were added at 2 µg mL^−1^ for 30 min at RT. For both trials, additional sets of high-binding 384-well plates were coated with 0.5 µg mL^−1^ of polyvalent goat anti-human Ig Ab (Life Technologies, Cat# H17000) to measure IgG, IgA, and IgM levels. All coating reagents were diluted in 0.1 M NaHCO_3_ solution. After washing, culture supernatants were added at 1:3 dilution in blocking buffer and incubated for 2 h at RT. After two washes with PBS/0.1% Tween-20, secondary goat anti-human IgG, anti-IgA and anti-IgM HRP-conjugated antibodies (Jackson ImmunoResearch, Cat. no. 109-035-098, 109-035-011, and 109-035-129, respectively) were added at lot-specific optimal concentrations for 1 h. After 4 washes, plates were developed for 10 min using 15 µL per well SureBlue Reserve TMB microwell peroxidase substrate (KPL) pre-equilibrated at RT. Development was stopped after 10 min with of 0.1 M HCl (15 µL/well). Plates were read at 450 nm and 640 nm (for background subtraction) wavelengths in a SpectraMax 384PLUS reader (Molecular Devices). All of the following criteria had to be met to define culture supernatant positivity for binding to Env immunogens: measurable IgG, IgA, or IgM levels; OD_450_  >  0.1 and > 3 × OD_450_ reads from blank wells; OD_450_  >  120% OD_650_.

### HIV-1 gp120 Env phylogenetic analysis

The unrooted phylogeny of the four HIV-1 gp120 protein sequences was calculated using the maximum likelihood algorithm implemented in IQtree-2. The same topology with similar branch lengths was also calculated using the Bayesian phylogenetic analysis program MrBayes.

### Isolation of immunoglobulin V_H_DJ_H_ gene segments

RNA from positive cultures was extracted using standard procedures (RNeasy minikit; Qiagen), and the genes encoding Ig V_H_DJ_H_ rearrangements were amplified by RT and nested PCR as previously reported^15,16^. Reverse transcription was performed at 50 °C for 1 h, 55 °C for 30 min, and 75 °C for 15 min after the addition of 200U/reaction of Superscript III RT (Life Technologies) and random hexamers (Gene Link). After cDNA synthesis, V_H_ genes were amplified by two rounds of PCR in 50 μL reaction mixtures. The first-round used 5 μL cDNA, 25 μL AmpliTaq Gold 360 master mix, 0.5 μM nested constant-region primers (IgH consisting of IgM, IgD, IgG, IgA1 and IgA2), and matched variable-region primers; in the second round, 3 μl first-round reaction product, 25 μL AmpliTaq Gold 360 master mix, 0.5 μM nested constant-region and nested variable-region primers were used. Both rounds of PCR were cycled as follows: 95 °C for 5 min; 35 cycles at 94 °C for 30 s, 62 °C for 45 s, and 72 °C for 90 s; and 1 cycle at 72 °C for 7 minutes. PCR products were analyzed on 2% agarose gels (Invitrogen). Antibody sequences were analyzed using a custom-built bioinformatics pipeline for base-calling, contig assembly, quality trimming, immunogenetic annotation with Cloanalyst (https://www.bu.edu/computationalimmunology/research/software/), V_H_DJ_H_ sequence quality filtering, and functionality assessment.

### Statistics and reproducibility

A two-tailed Mann–Whitney U test was performed to determine significance of differences in somatic hypermutation levels, a continuous variable, between two groups. Since individual datapoints were not matched between groups, we used an unpaired statistical test. We used a nonparametric test to avoid the assumption of Gaussian distribution of the sampled populations. Analyses were performed using GraphPad Prism (version 10).

### Reporting summary

Further information on research design is available in the Nature Portfolio Reporting Summary linked to this article.

## Results

### B cell cross-reactivity upon immunization with a multivalent, multiclade gp120 Env glycoprotein formulation

**Fig. 1:**
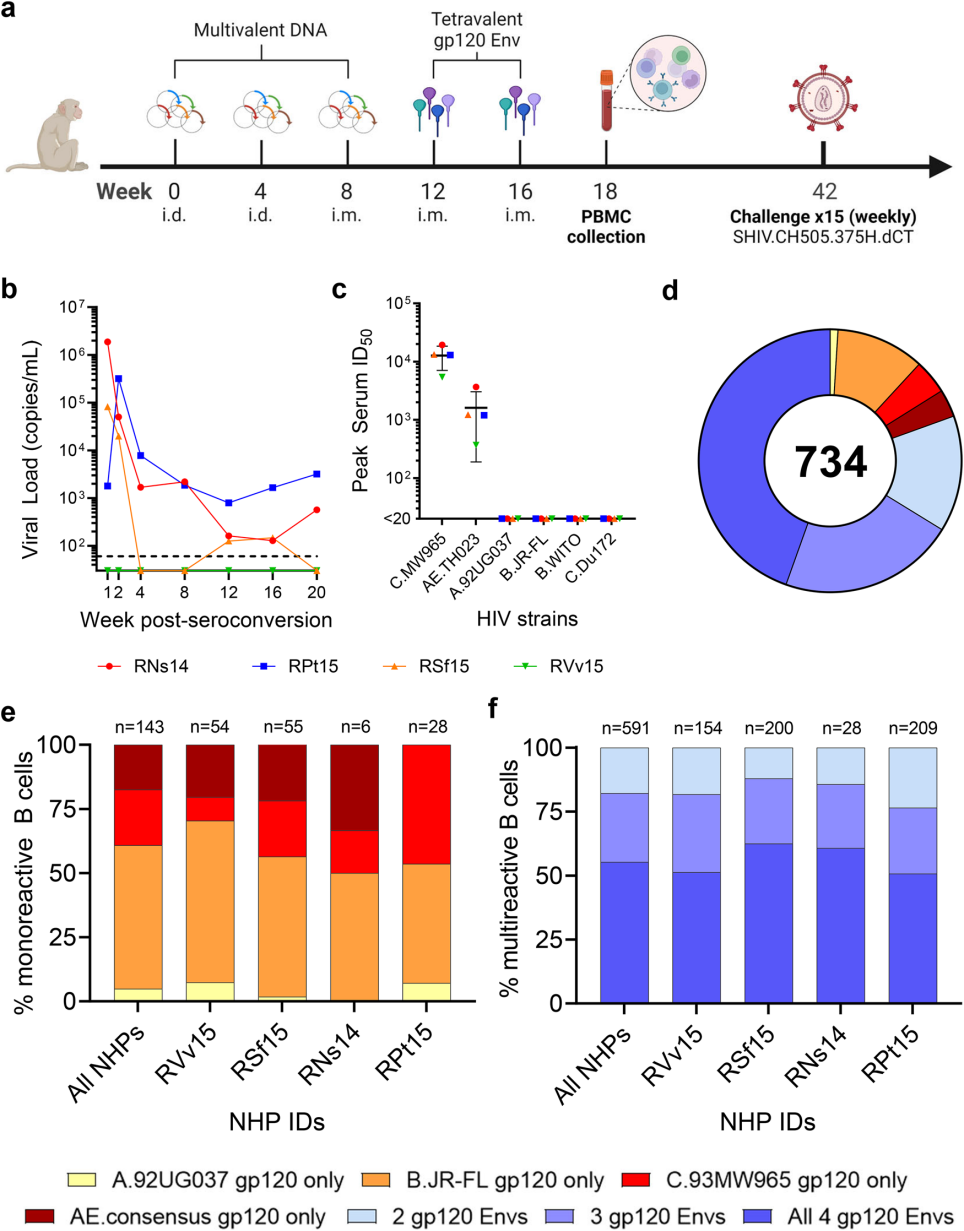
Tetravalent gp120 Env immunization. **a** NHP immunization scheme with multivalent HIV-1 gp120 Env cocktails delivered as DNA and monomeric proteins. i.d. intradermal, i.m. intramuscular. **b** Viremia time-course of the four NHPs included in this study post-seroconversion or after the fifteenth challenge for RVv15. Each NHP is color-coded as follows: red circles: RNs14; blue squares: RPt15; orange triangles: RSf15; green inverted triangles: RSf15. Dashed line at assay limit of sensitivity threshold (60 copies/ml). Measurements were performed in technical duplicates. Also see Supplementary Data 1. **c** Serum was collected four weeks prior to immunization and again at weeks 10 and 18 post-immunization. Peak serum neutralization data, defined as highest ID_50_ value within the time course, are shown for the four NHPs in this study against two tier 1 viruses (C.MW965.26 and TH023.6) and four tier 2 viruses (A.92UG037.1, B.JR-FL, B.WITO4160.33, and C.Du172.17). Each NHP is color-coded as indicated in panel b. Lines indicate group mean and standard deviation. Measurements were performed in technical duplicates. Also see Supplementary Data 2. **d** Immunogen binding profiles of 734 B cells isolated from all four NHPs. Reactivity with individual and multiple gp120 Env immunogens is color coded as indicated: yellow: A.92UG037 gp120 only; orange: B.JR-FL gp120 only; red: C.93MW965 gp120 only; brown: AE.consensus gp120 only; light blue: 2 gp120 Envs; violet: 3 gp120 Envs; dark blue: all 4 gp120 Envs. Also see Supplementary Data 3. **e** Binding to individual immunogens of monoreactive B cells, expressed as percentage of the total number of monoreactive B cells. Data are shown as aggregate (“All NHPs”) and for each NHP. Reactivity with individual and multiple gp120 Env immunogens is color coded as indicated in panel d. Also see Supplementary Data 4. **f** Immunogen cross-reactivity of multireactive B cells, expressed as percentage of the total number of multireactive B cells, shown as aggregate (“All NHPs”) and for each NHP. Reactivity with individual and multiple gp120 Env immunogens is color coded as indicated in panel d. Also see Supplementary Data 5.

In the first study, four female macaques (RNs14, RVv15, RPt15 and RSf15) were primed with DNA and boosted with a tetravalent HIV-1 gp120 Env cocktail comprising clades A, B, C and CRF AE strains (Fig. 1a). Upon heterologous SHIV challenge, all but one macaque (RVv15) seroconverted (Fig. 1b). RNs14 and RPt15 seroconverted at the second challenge, and RSf15 seroconverted at the fourth challenge. Comparatively, two of ten NHPs in the mock control group also resisted infection. Serum neutralization was tested against tier 1AC.MW965.26 and AE.TH023.6 strains, and tier 2A.92UG037.1, B.JR-FL, B.WITO4160.33 and C.Du172.17 strains. All macaques neutralized the easy-to-neutralize tier 1A strains, with a geometric mean peak serum ID_50_ of 11,599 for C.MW965.26 and 1186 for AE.TH023.6 (Fig. 1c). Memory B cells that bound to the tetravalent cocktail of Env proteins were sorted from PBMCs collected two weeks post-final boost and cultured as described^12^. Across all NHPs, immunogen-reactive B cells constituted 0.5–2% of the circulating memory B cells (RPt15: 1.31%, RNS14: 0.77%, RSf15: 2.04%, RVv15: 0.52%) (Supplementary Fig. S2), which is in line with the frequency we previously reported for a human trial that used bivalent recombinant gp120 immunogens^17^. For each B cell culture supernatant, binding to individual immunogens was assessed. In total, we analyzed 734 Env-reactive B cells (RNs14: *n* = 34, RVv15: *n* = 208, RPt15: *n* = 237, RSf15: *n* = 255). Only 143/734 (19%) reacted with a single Env immunogen (Fig. 1d). Mono-specificity was predominantly directed to B.JR-FL (*n* = 80, 56%), followed by C.93MW965 (*n* = 31, 22%), AE consensus (*n* = 25, 17%), and A.92UG037 (*n* = 7, 5%), with similar distributions across the four macaques (Fig. 1e). Of the 591 B cells that bound to multiple Envs, the majority (*n* = 327/591; 55%) reacted with all four immunogens (RNs14: *n* = 17/28, 61%; RVv15: *n* = 79/154, 51%; RPt15: *n* = 106/209, 51%; RSf15: *n* = 125/200, 63%) (Fig. 1f). Thus, the B cells expanded by this tetravalent multiclade formulation predominantly converged toward epitopes shared among the immunogens.

### B cell cross-reactivity upon sequential immunization with non-stabilized and SOSIP-stabilized gp140 Env glycoproteins

**Fig. 2:**
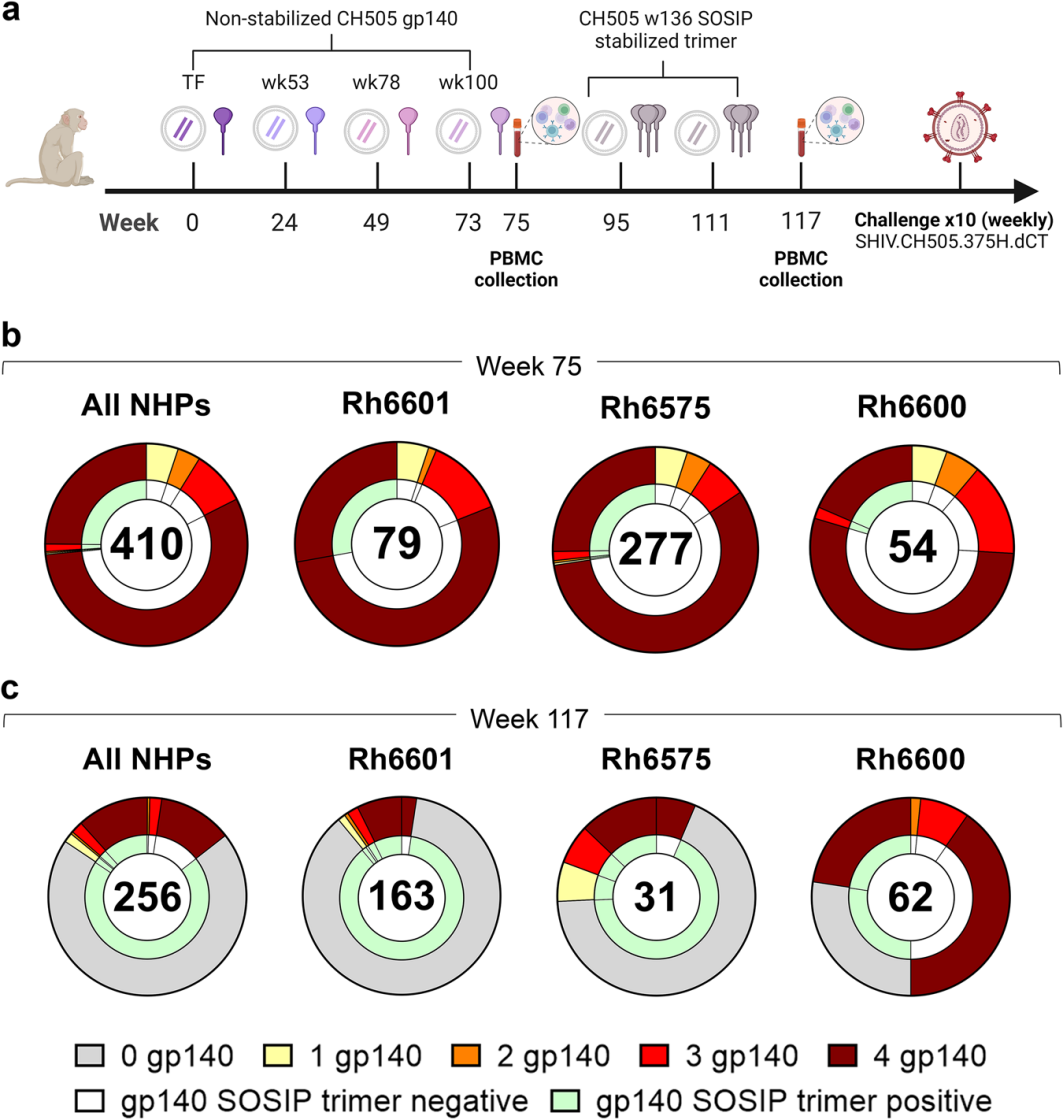
Sequential immunization with clonally related Envs. **a** NHP immunization scheme (see methods and ref. ^9^). TF transmitted founder. **b, c** Cross-reactivity of B cells with non-stabilized gp140 Envs immunogens (outer ring) and stabilized CH505.w136 SOSIP trimer (inner ring) (**b**) before and (**c**) after CH505.w136 SOSIP trimer immunizations. Data are shown as aggregate (“All NHPs”) and for each NHP. Number of B cells analyzed is shown for each chart. Reactivity with individual and multiple non-stabilized and SOSIP stabilized gp140 Env immunogens is color coded as follows: gray: no reactivity with non-stabilized gp140 Envs (0 gp140); yellow: reactivity with one non-stabilized gp140 Env; orange: reactivity with two non-stabilized gp140 Envs; red: reactivity with three non-stabilized gp140 Envs; brown: reactivity with four non-stabilized gp140 Envs; white: no reactivity with SOSIP-stabilized gp140 Env; green: reactivity with SOSIP-stabilized gp140 Env. Also see Supplementary Data 6 and 7.

In the second study, macaques were immunized sequentially with gp140 Envs isolated from individual CH505 (transmitted founder virus [TF] and variants isolated from weeks 53, 78, 100 and 136 post-infection)^9^. Immunogens were delivered via an integrase-defective lentiviral vector (IDLV) either alone or with the respective protein (Fig. 2a)^9^. The first four immunogens were administered as non-stabilized gp140 Envs, whereas CH505.wk136 was administered as SOSIP-stabilized gp140 Env trimer intended to focus antibody responses toward broadly neutralizing epitopes^9^. SOSIP-stabilized trimers had been engineered to stabilize the native-like trimeric conformation of soluble gp140 Env by introducing an intramolecular disulfide bond (SOS) to link the gp120 and the gp41 ectodomain subunits and the I559P point mutation (IP) in the gp140 ectodomain to maintain them in the pre-fusion conformation^18^. As we previously reported, no neutralization against the tier 2 viruses autologous to the vaccine or the challenge strain (SHIV CH505.375H) was observed upon completion of the immunization regimen^9^. Upon repeated autologous SHIV challenge, all but one macaque (Rh6601, male, IDLV+protein group) were infected, with two macaques (Rh6600, male, IDLV+protein group and Rh6575, female, IDLV only group) resisting infection up to the tenth challenge^9^. In these three macaques, we measured the frequency of Env immunogen-specific memory B cell responses after the last non-stabilized gp140 Env and the SOSIP-stabilized gp140 Env administrations (weeks 75 and 117, respectively). Across all NHPs, immunogen-reactive B cells constituted 0.4–2.7% of the circulating memory B cells at week 75 (Supplementary Fig. S3a), but only 0.09–0.6% at week 117 (Supplementary Fig. S3b).

From week 75, we analyzed a total of 410 Ig+ culture supernatants from immunogen-reactive B cells (Rh6601: *n* = 79, Rh6600: *n* = 54, Rh6575: *n* = 277). Most B cells (*n* = 331/410, 81%) cross-reacted with all gp140 Env immunogens (Rh6601: *n* = 64/79, 81%; Rh6600: *n* = 39/54, 72%; Rh6575: *n* = 228/277, 82%) and <6% reacted with a single gp140 Env (Rh6601: *n* = 4, 5.1%; Rh6600: *n* = 3, 5.6%; Rh6575: *n* = 15, 5.4%) (Fig. 2b and Supplementary Fig. S4). Among the gp140 Env-reactive B cells, 27% also reacted with the CH505.wk136 SOSIP-stabilized trimer (Rh6601: *n* = 22/79, 28%; Rh6600: *n* = 11/54, 20%; Rh6575: *n* = 76/277, 27%), which was not yet administered at week 75 (Fig. 2b). The vast majority (95%) of the SOSIP-stabilized trimer-binding B cells reacted with all four gp140 Env immunogens (Fig. 2b). Hence, sequential immunization with non-stabilized gp140 Envs expanded B cells that cross-reacted with and were available for engagement to the CH505.wk136 SOSIP-stabilized trimer immunogen. In line with the multivalent, multiclade study described above, these results demonstrate that immunization with clonally related gp140 Envs elicited a dominant B cell response that converged toward epitopes shared across the immunogens.

After CH505.wk136 SOSIP-stabilized trimer boosting (week 117), trimer-reactive cells increased from 27% to 86% (All NHPs: 219/256; Rh6601: 159/163, 98%; Rh6600: 31/62, 50%; Rh6575: 29/31, 94%) (Fig. 2c). We note that the CH505.wk136 SOSIP-stabilized trimer ELISA for Rh6600 had high background, which likely resulted in an underestimate of trimer-reactive cultures for this macaque. Differently from what was observed after immunization with non-stabilized gp140 Envs, the majority of CH505.wk136 SOSIP-stabilized trimer-reactive B cells (179/219, 82%) did not cross-react with the preceding gp140 Env immunogens (Rh6601: 141/159, 89%; Rh6600: 17/31, 55%; Rh6575: 21/29, 72%) (Fig. 2c).

**Fig. 3:**
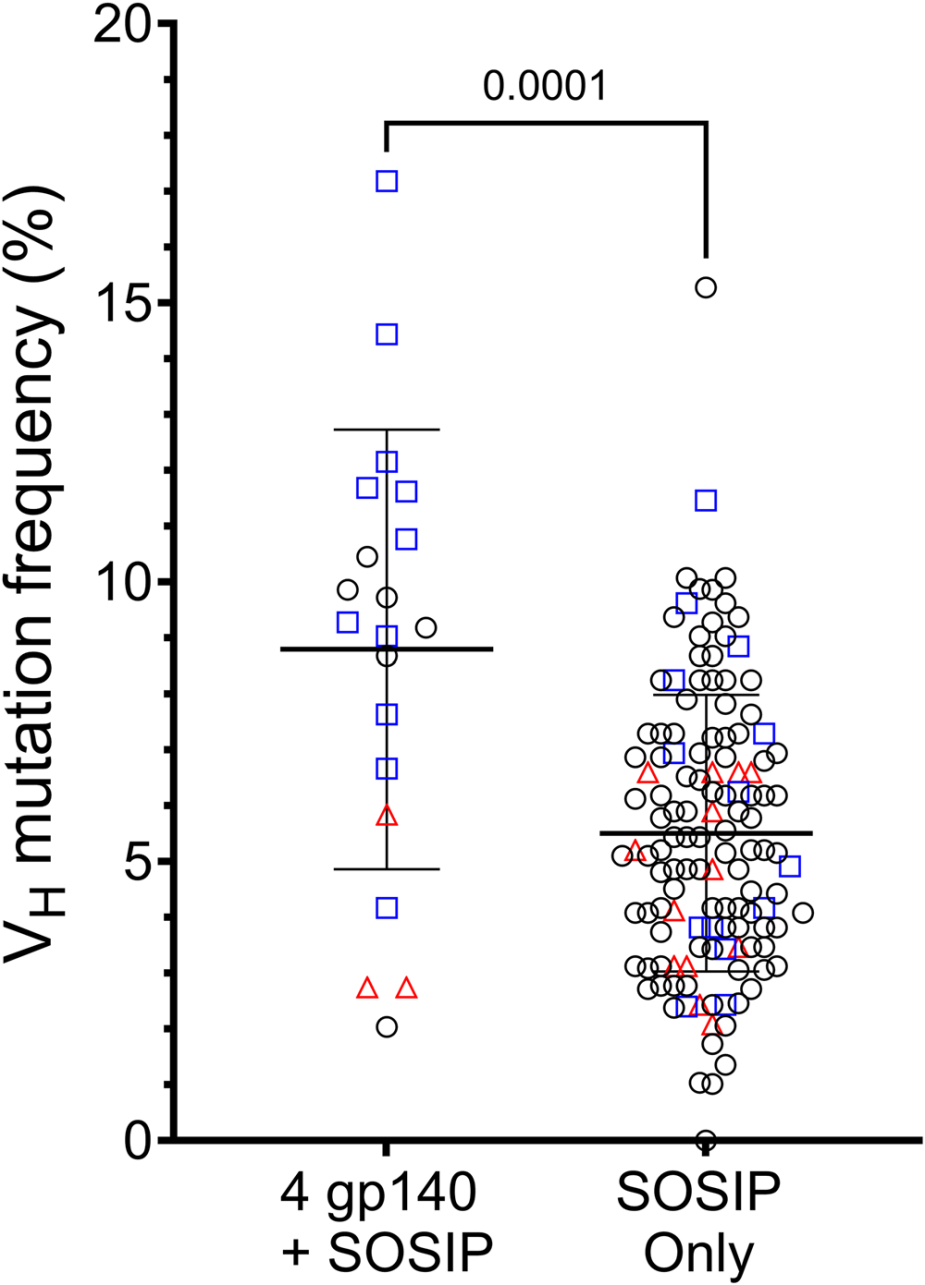
Somatic hypermutation frequencies of IgV_H_ gene segments isolated from memory B cells collected after the end of the sequential immunization with IDLV expressing CH505 Envs. We analyzed a total of 150 IgV_H_DJ_H_ sequences from memory B cells isolated at week 117 that bound to either all immunogens (*n* = 20, left column) or to the CH505.wk136 SOSIP-stabilized trimer immunogen only (*n* = 130, right column). IgV_H_ gene segment somatic hypermutation frequencies (y-axis) were calculated using Cloanalyst (see methods). Lines show the mean and standard deviation for each group. Statistically significant difference between the two groups (*p* = 0.0001) was evaluated using the non-parametric, two-tailed Mann-Whitney test. The NHP source for each sequence is coded as follows: red triangles: Rh6575; blue squares: Rh6600; black circles: Rh6601. Also see Supplementary Data 8 and 9.

To assess the maturation levels of antibodies with the most extreme differential reactivity profiles, we sequenced IgV_H_DJ_H_ rearrangements of immunogen-specific memory B cells isolated from all three NHPs at week 117 and compared the V_H_ somatic hypermutation frequencies (SHM) of antibodies that bound to all immunogens (*n* = 20) versus those that bound only to the CH505.wk136 SOSIP-stabilized trimer (*n* = 130). The V_H_ of CH505.wk136 SOSIP-only binders were significantly less mutated than those of the cross-reactive antibodies (8.8% vs 5.5%; *p* = 0.0001, two-tailed Mann–Whitney test) (Fig. 3).

All data taken together, we conclude that the change in immunogen design from non-stabilized gp140 to SOSIP-stabilized trimeric Env induced the engagement of a divergent subset of B cells.

## Discussion

In summary, this study exemplifies how the choice of immunogen designs can affect the engagement of different B cell pools in the context of multivalent and sequential immunizations: Specifically, how they can alter the balance between B cells that cross-react with multiple immunogens (convergent response) and those that instead elicit a divergent response that vastly fails to engage prior immunogens in the series. Despite the genetic distance among immunogens in the multiclade immunization scheme (Supplementary Fig. S5), the observed cross-clade binding was predominantly mediated by B cells that converged on epitopes shared among the immunogens, rather than by an additive engagement of multiple clade-specific B cells. Convergence was unsurprisingly more pronounced in sequential immunizations with clonally related non-stabilized gp140 Envs. However, subsequent SOSIP stabilized gp140 Env immunization mostly engaged less somatically mutated B cells that failed to cross-react with the preceding gp140 Env immunogens and were present at lower frequency in peripheral blood.

Non-stabilized gp140 Envs are known to expose non-neutralizing epitopes in regions that are masked on stabilized trimers^19^. Nonetheless, non-stabilized gp140 Env immunization expanded a pool of B cells capable of binding to the SOSIP-stabilized gp140 trimers. Therefore, epitope masking cannot be invoked to explain SOSIP failure to primarily expand such cross-reactive population. The implication is that the epitope immunodominance hierarchy of the SOSIP trimer differed from that of the non-stabilized gp140 Envs, and that a different set of B cells was primarily engaged (divergent response). SOSIP-stabilized trimers are indeed used as a polishing step to favor the selective maturation of neutralizing antibody responses (reviewed in refs. ^20,21^). However, we had previously shown that this sequential immunization regimen did not yield tier 2 neutralization, including the autologous CH505 transmitted founder strain, or the development of CD4bs bnAb precursors, despite two CD4bs bnAb lineages (CH103 and CH235) were isolated from the CH505 individual^9,22–24^. Therefore, we did not observe the expected polishing effect. Rather, our findings are in line with recent observations that the BG505 SOSIP.664 trimer backbone, which was used as template for this study, displays a dominant neo-epitope cluster at the base region of the trimer^25–28^. Critically, and regardless of the specific epitopes involved, we demonstrate that, in this regimen, B cell priming with non-stabilized Env was insufficient to modify the effect of a different epitope immunodominance hierarchy in a SOSIP trimer. Conversely, the change in format of the immunogen was sufficient to induce a switch from B cell responses that converged on epitopes conserved across the immunogens to responses that mostly diverged, despite conserved epitopes being accessible.

Immunogen engineering is a powerful tool to alter immunodominance hierarchies^29^. These results suggest that caution is needed to avoid off-target B cell engagement when changing immunogen designs within sequential immunizations. In particular, immunodominance hierarchies of epitopes presented on different designs may not be similar. This consideration is also in principle relevant to hybrid immunogens targeting multiple viruses (e.g., influenza and SARS-CoV-2) and polyvalent preparations aimed at inducing balanced responses against multiple virus serotypes (e.g., Dengue virus). These results also suggest that, should B cell repriming be desirable (e.g., to target immunodominant epitopes introduced in highly mutating viruses through natural evolution) pre-existing immunity against other epitopes should not constitute an insurmountable obstacle.

## Supplementary information

Supplementary information

Description of Additional Supplementary Files

Supplementary Data 1

Supplementary Data 2

Supplementary Data 3

Supplementary Data 4

Supplementary Data 5

Supplementary Data 6

Supplementary Data 7

Supplementary Data 8

Supplementary Data 9

Supplementary Data 10

Reporting Summary

## Data Availability

All source data underlying the Figs. 1–3 and Supplementary Fig. S5 are provided in Supplementary Data 1–10. Sequences are deposited in GenBank with accession numbers PV221792 through PV221941. The datasets generated and analyzed in this study are available from the corresponding author on reasonable request.

## Abbreviation list

Env: envelope glycoprotein
NHP: non-human primate
PBMC: peripheral blood mononuclear cell
SHIV: Simian-Human Immunodeficiency Virus
bnAb: broadly neutralizing antibody
