# Supplementary material for: Convergence and divergence of B cell responses in two HIV-1 Env immunizations in Rhesus macaques
Source: Commun Med (Lond). 2025 May 15;5:175. doi: 10.1038/s43856-025-00899-3 (PMC12081853; doi:10.1038/s43856-025-00899-3)
Supplement: Supplementary file 1 — Supplementary information [file 43856_2025_899_MOESM1_ESM.pdf]

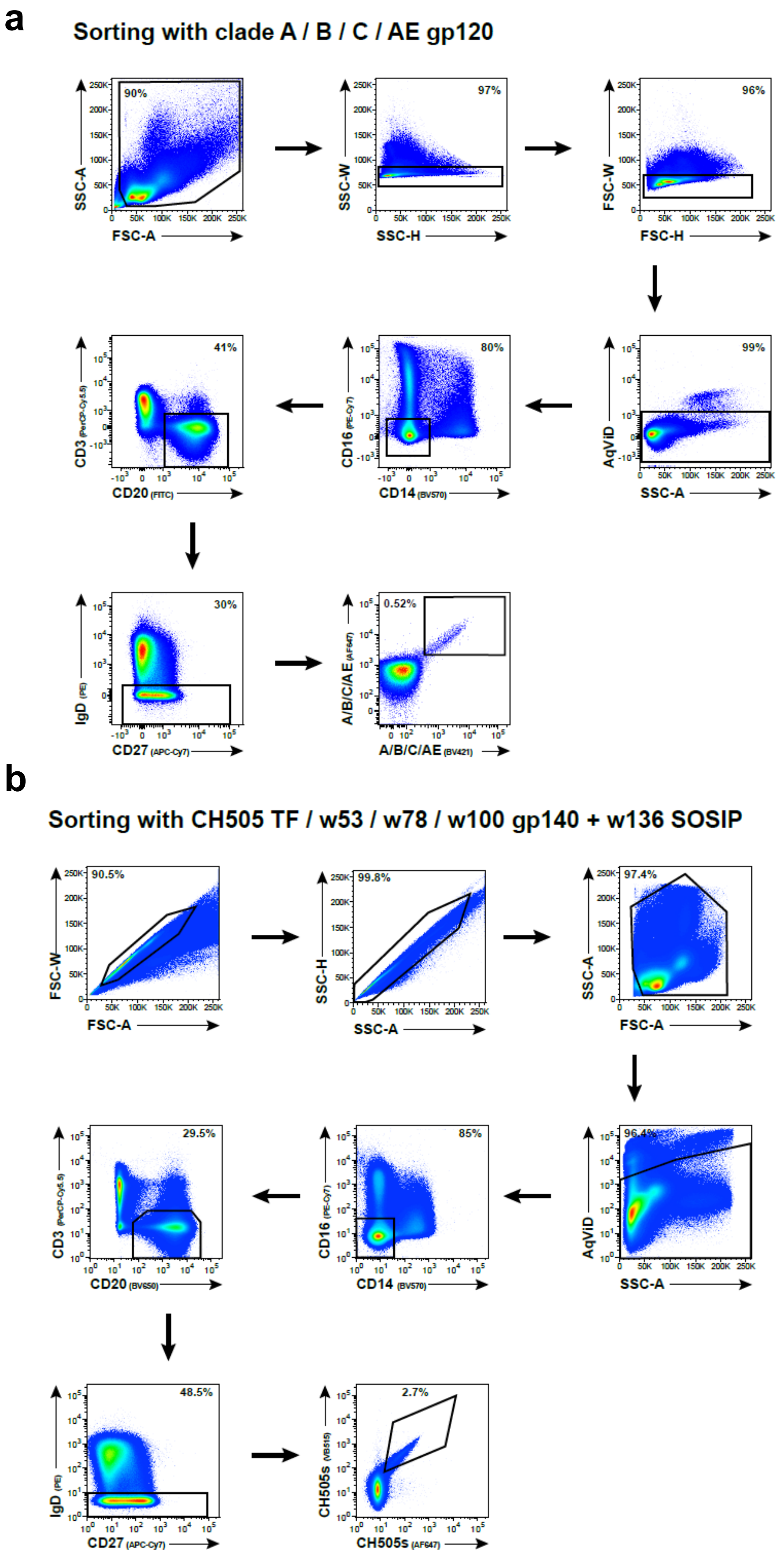

Figure S1. Representative flow cytometry sort plots of immunogen specific memory B cells. PBMC isolated from macaques were stained, and immunogen-specific memory B cells were sorted. Cells were gated based on viability, negative selection of non-B cell markers (CD3, CD14, CD16), and positive selection for CD20. Memory B cells were defined as surface IgD negative, then cells were gated for double positive reactivity with fluorochrome-labeled gp120 **(a)** or gp140 **(b)** Envs.

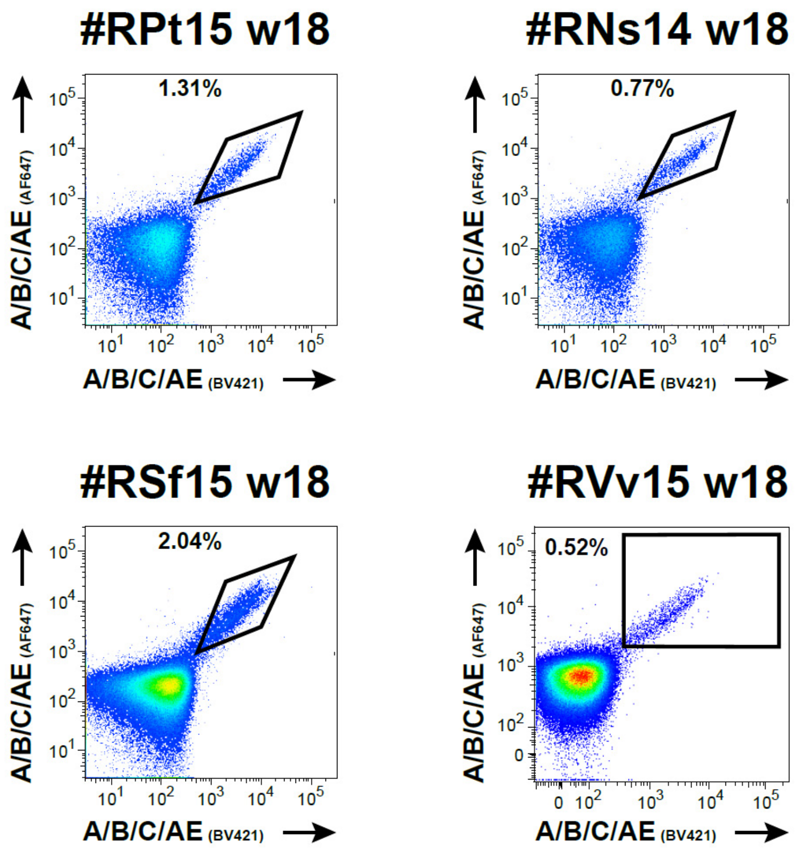

Figure S2. Frequency of immunogen-reactive memory B cells isolated from four NHPs immunized with a multivalent, multiclade Env formulation. Flow plots of A.92UG037.1, B.JR-FL, C.93MW965.26 and CRF AE consensus gp120 Env staining of memory B cells isolated 2 weeks after the last immunization (week 18, as reported in Figure 1) from NHPs RPt15, RNs14, RSf15 and RVv15. Memory B cells that bound to BV421 (x-axis) and AF647 (y-axis) labeled Envs were defined as double positive (DP) Env-reactive memory B cells and measured to determine the immunogen-reactive memory B cell frequencies. Sorting of DP Env-reactive B cells was performed to ensure sort accuracy.

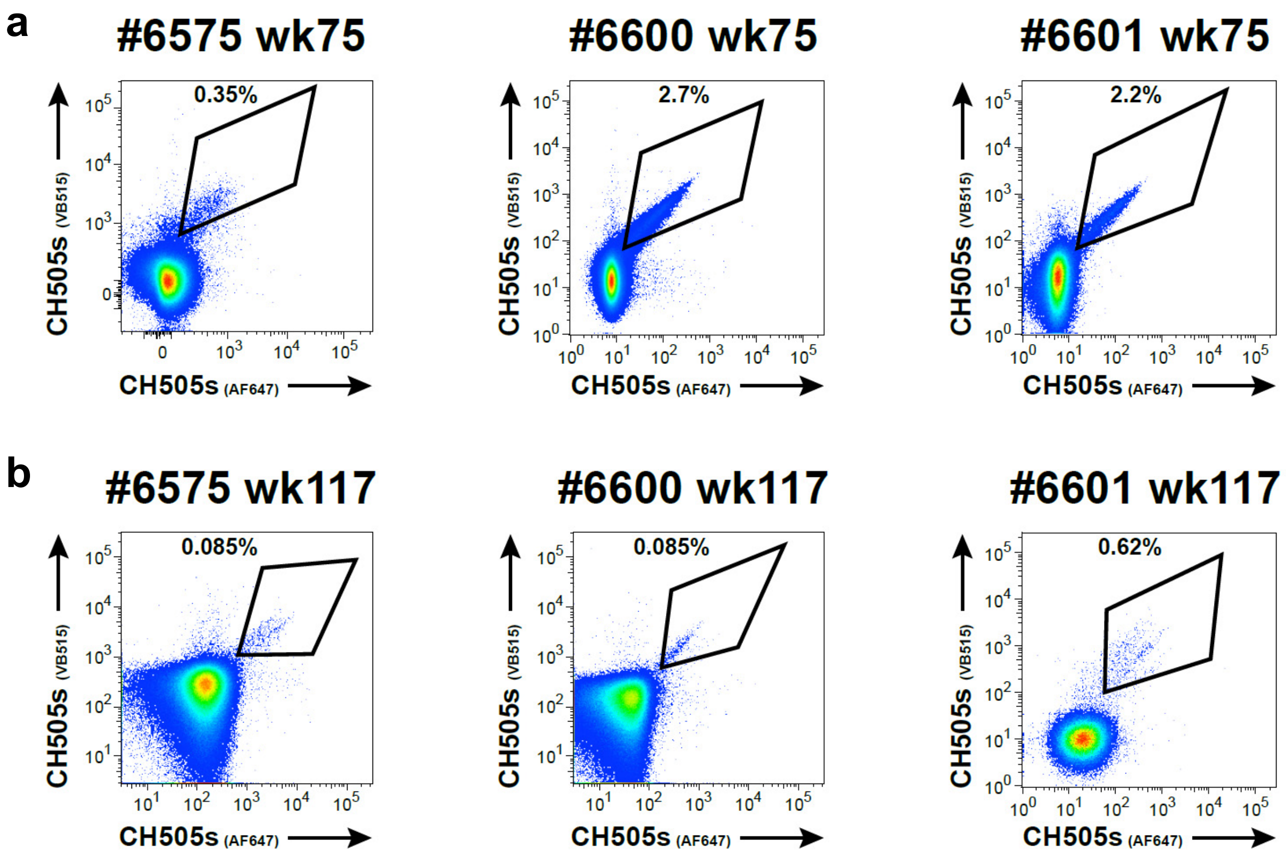

Figure S3. Frequency of immunogen-reactive memory B cells isolated from three NHPs sequentially immunized with IDLV expressing CH505 Envs. Flow plots of CH505.TF, CH505.wk53, CH505.wk78 and CH505.wk100 non-stabilized gp140 Envs, and SOSIP-stabilized CH505.wk136 gp140 Env staining of memory B cells isolated from NHPs 6575, 6600 and 6601 **(a)** two weeks after the last non-stabilized gp140 Env immunization (week 75), or **(b)** six weeks after the last SOSIP-stabilized CH505.wk136 gp140 Env immunization (week 117), as reported in Figure 2. Memory B cells that bound to AF647 (x-axis) and VB515 (y-axis) labeled Envs were defined as double positive (DP) Env-reactive memory B cells and measured to determine the immunogen-reactive memory B cell frequencies. Sorting of DP Env-reactive B cells was performed to ensure sort accuracy.

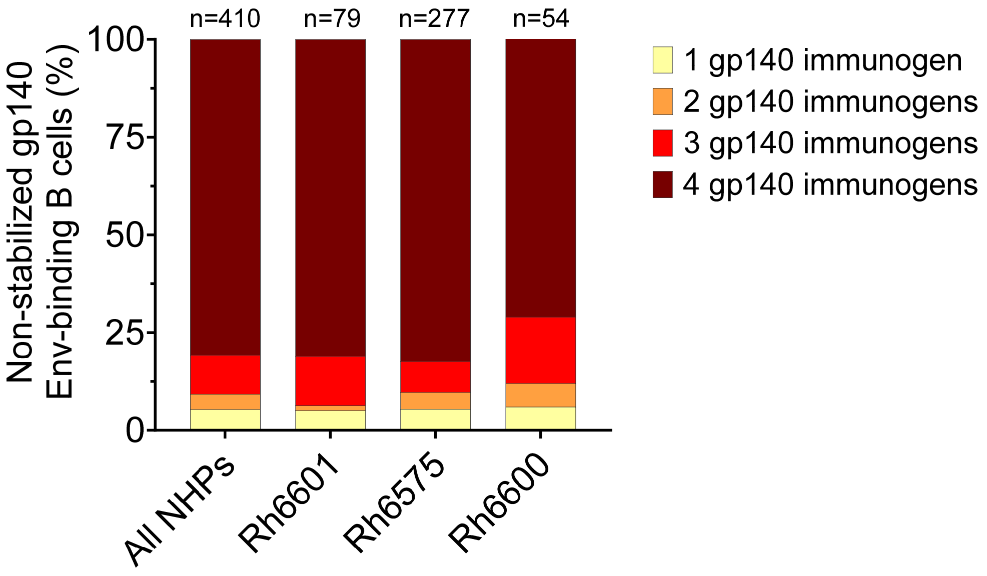

Figure S4. Binding cross-reactivity profile to non-stabilized gp140 Env immunogens of B cells isolated before SOSIP trimer immunization (week 75). The number of non-stabilized gp140 Env immunogens bound by each B cell is colored coded as shown on the right. Results are expressed as percentage relative to the total number of B cells analyzed. Data are shown in aggregate form in the "All NHPs" column and for each individual NHP as indicated.

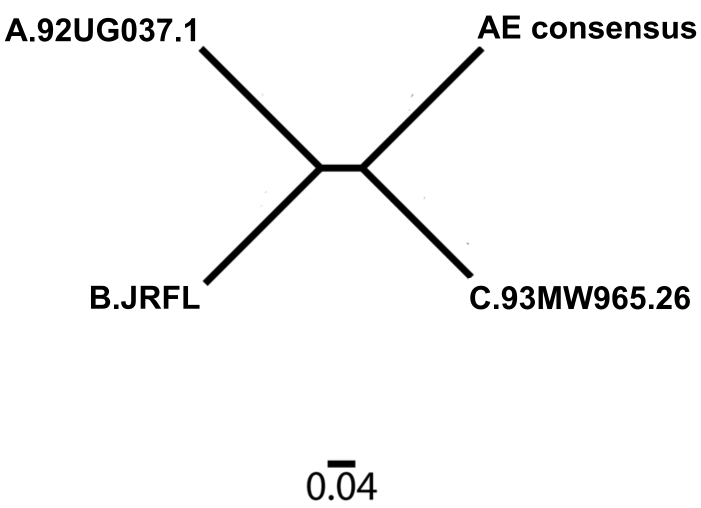

Figure S5. Unrooted phylogeny of the gp120 Env protein included in the tetravalent boost. Distance among the four gp120 Envs included in the tetravalent boost of the multiclade, multivalent immunization regimen, calculated from gp120 Env amino acid sequences of each immunogen. Each immunogen is identified with its clade and strain name. Tree was calculated using the maximum likelihood algorithm implemented in IQtree-2, as reported in the methods. Also see Supplementary Data 10.
