# Supplementary material for: Convergence and divergence of B cell responses in two HIV-1 Env immunizations in Rhesus macaques
Source: Commun Med (Lond). 2025 May 15;5:175. doi: 10.1038/s43856-025-00899-3 (PMC12081853; doi:10.1038/s43856-025-00899-3)
Supplement: Supplementary file 2 — Description of Additional Supplementary Files [file 43856_2025_899_MOESM2_ESM.pdf]

Description of Additional Supplementary Files

File name: Supplementary Data 1

File description: Data underlying the viremia time course shown in Figure 1b.

File name: Supplementary Data 2

File description: Data underlying the peak serum neutralization data shown in Figure 1c.

File name: Supplementary Data 3

File description: Data underlying the immunogen binding profiles in Figure 1d.

File name: Supplementary Data 4

File description: Data underlying the monoreactive B cell binding profile shown in Figure 1e.

File name: Supplementary Data 5

File description: Data underlying the multireactive B cell binding profile shown in Figure 1f.

File name: Supplementary Data 6

File description: Data underlying week 75 binding profile shown in Figure 2b.

File name: Supplementary Data 7

File description: Data underlying week 117 binding profile shown in Figure 2c.

File name: Supplementary Data 8

File description: Data underlying somatic hypermutation data shown in Figure 3.
