# Supplementary material for: Convergence and divergence of B cell responses in two HIV-1 Env immunizations in Rhesus macaques
Source: Commun Med (Lond). 2025 May 15;5:175. doi: 10.1038/s43856-025-00899-3 (PMC12081853; doi:10.1038/s43856-025-00899-3)
Supplement: Supplementary file 13 — Reporting Summary [file 43856_2025_899_MOESM13_ESM.pdf]

nature portfolio

Corresponding author(s): Mattia Bonsignori

Last updated by author(s): 8/21/2024

Reporting Summary

Nature Portfolio wishes to improve the reproducibility of the work that we publish. This form provides structure for consistency and transparency in reporting. For further information on Nature Portfolio policies, see our Editorial Policies and the Editorial Policy Checklist.

Statistics

For all statistical analyses, confirm that the following items are present in the figure legend, table legend, main text, or Methods section.

n/a	Confirmed	
☐	☒	The exact sample size (*n*) for each experimental group/condition, given as a discrete number and unit of measurement
☐	☒	A statement on whether measurements were taken from distinct samples or whether the same sample was measured repeatedly
☐	☒	The statistical test(s) used AND whether they are one- or two-sided<br>*Only common tests should be described solely by name; describe more complex techniques in the Methods section.*
☒	☐	A description of all covariates tested
☒	☐	A description of any assumptions or corrections, such as tests of normality and adjustment for multiple comparisons
☐	☒	A full description of the statistical parameters including central tendency (e.g. means) or other basic estimates (e.g. regression coefficient) AND variation (e.g. standard deviation) or associated estimates of uncertainty (e.g. confidence intervals)
☒	☐	For null hypothesis testing, the test statistic (e.g. *F*, *t*, *r*) with confidence intervals, effect sizes, degrees of freedom and *P* value noted<br>*Give P values as exact values whenever suitable.*
☒	☐	For Bayesian analysis, information on the choice of priors and Markov chain Monte Carlo settings
☒	☐	For hierarchical and complex designs, identification of the appropriate level for tests and full reporting of outcomes
☒	☐	Estimates of effect sizes (e.g. Cohen's *d*, Pearson's *r*), indicating how they were calculated

Our web collection on statistics for biologists contains articles on many of the points above.

Software and code

Policy information about availability of computer code

Data collection	SoftMax Pro 7 and BD FACSDiva 8.0.1
Data analysis	SoftMax Pro 7, Cloanalyst, GraphPad Prism, Microsoft Excel, MrBayes, and IQtree-2

For manuscripts utilizing custom algorithms or software that are central to the research but not yet described in published literature, software must be made available to editors and reviewers. We strongly encourage code deposition in a community repository (e.g. GitHub). See the Nature Portfolio guidelines for submitting code & software for further information.

Data

Policy information about availability of data

All manuscripts must include a data availability statement. This statement should provide the following information, where applicable:

- Accession codes, unique identifiers, or web links for publicly available datasets
- A description of any restrictions on data availability
- For clinical datasets or third party data, please ensure that the statement adheres to our policy

The datasets generated and analyzed in this study are available from the corresponding author on reasonable request.

Human research participants

Policy information about studies involving human research participants and Sex and Gender in Research.

Reporting on sex and gender	No human research participants were involved in this study.
Population characteristics	No human research participants were involved in this study.
Recruitment	No human research participants were involved in this study.
Ethics oversight	No human research participants were involved in this study.

Note that full information on the approval of the study protocol must also be provided in the manuscript.

Field-specific reporting

Please select the one below that is the best fit for your research. If you are not sure, read the appropriate sections before making your selection.

☒ Life sciences ☐ Behavioural & social sciences ☐ Ecological, evolutionary & environmental sciences

For a reference copy of the document with all sections, see nature.com/documents/nr-reporting-summary-flat.pdf

Life sciences study design

All studies must disclose on these points even when the disclosure is negative.

Sample size	All samples used in this study were obtained from pre-existing cohorts of Rhesus macaques from two independent studies. As described in the manuscript, the selection criteria and number of non-human primates (NHP) included in this report were as follows: For immunizations with the multivalent, multiclade regimen, macaques were challenged up to 15 times and we selected three macaques that seroconverted early and one macaque that resisted challenge. For sequential immunizations with clonally related Envs, macaques were challenged up to 10 times and we selected the three macaques that resisted infection at least up to the 10th challenge.
Data exclusions	All of the following pre-established criteria had to be met to define culture supernatant positivity for binding to Env immunogens: measurable IgG, IgA, or IgM levels; $OD450>0.1$ and $>3\times OD450$ reads from blank wells; $OD450>120\%$ OD650.
Replication	The supernatant screenings on reactivity of the primary limiting-diluted sorted B cells was performed in singletons due to the limited amount of available supernatant. Every 96-well assay plate included a serially diluted positive control, culture supernatants negative for Ig production and blank wells to validate the assay.
Randomization	Not relevant, see criteria above.
Blinding	Blinding was not relevant to this study because positivity criteria (listed above) were pre-established.

Reporting for specific materials, systems and methods

We require information from authors about some types of materials, experimental systems and methods used in many studies. Here, indicate whether each material, system or method listed is relevant to your study. If you are not sure if a list item applies to your research, read the appropriate section before selecting a response.

Materials & experimental systems

n/a	Involved in the study	
☐	☒	Antibodies
☐	☒	Eukaryotic cell lines
☒	☐	Palaeontology and archaeology
☐	☒	Animals and other organisms
☒	☐	Clinical data
☒	☐	Dual use research of concern

Methods

n/a	Involved in the study	
☒	☐	ChIP-seq
☐	☒	Flow cytometry
☒	☐	MRI-based neuroimaging

Antibodies

Antibodies used	CD20 FITC (BD Biosciences, catalog no. 347673, clone L27) or CD20 BV650 (BD Biosciences, catalog no. 740439, clone L27); CD3

Antibodies used	PerCP-Cy5.5 (BD Biosciences, catalog no. 552852, clone SP34-2), IgD PE (Southern Biotech, catalog no. 2030-09, polyclonal); CD8 PE-Texas Red (Invitrogen, catalog no. MHCD0817, clone 3B5); IgM PE-Cy5 (BD Biosciences, catalog no. 551079, clone G20-127); CD16 PE-Cy7 (BD Biosciences, catalog no. 557744, clone 3G8); CD27 APC-Cy7 (BioLegend, catalog no. 302816, clone O323); CD14 BV570 (BioLegend, catalog no. 301832, clone M5E2). Polyvalent goat anti-human Ig Ab (Life Technologies, Cat# H17000). Goat anti-human IgG, anti-IgA and anti-IgM HRP-conjugated antibodies (Jackson ImmunoResearch, Cat. no. 109-035-098, 109-035-011, and 109-035-129
Validation	All antibodies were titered in advance and lot-specific working concentrations were based on measured optimal signal-to-noise ratios.

Eukaryotic cell lines

Policy information about cell lines and Sex and Gender in Research

Cell line source(s)	MS40L cells (male mouse, stromal cell line) was kindly provided by Dr. David Baltimore, California Institute of Technology. IL-2t6 cells (myeloma cell line) was kindly provided by Dr. Antonio Lanzavecchia, IRB, Bellinzona, Switzerland.
Authentication	MS40L is a murine cell line derived from the stem cell line MS5 to express low levels of cell surface human CD40L. MS40L cells have been widely used to support robust B-cell growth in vitro in presence of additional stimulants and were used here as feeder cells for memory B cell cultures. The cell line was engineered by Dr. David Baltimore. Cell surface expression of key marker CD40L was routinely verified.<br><br>IL-2t6 cells were used as a source of human recombinant IL-2. Supernatants were collected from IL-2t6 cell cultures and IL-2 secretion was verified using a 21-plex human cytokine panel. IL-2 activity was tested on preliminary titration experiments to determine the lot-specific working dilution.
Mycoplasma contamination	Cells were routinely tested for mycoplasma.
Commonly misidentified lines (See ICLAC register)	No commonly misidentified lines were used in this study.

Animals and other research organisms

Policy information about studies involving animals; ARRIVE guidelines recommended for reporting animal research, and Sex and Gender in Research

Laboratory animals	Non-human primates: Rhesus macaques (Macaca mulatta)
Wild animals	This study did not involve wild animals.
Reporting on sex	Sex was not a variable considered in the selection of individual NHPs included in this study. Selection criteria are reported above.
Field-collected samples	This study did not involve samples collected from the field.
Ethics oversight	The Rhesus macaques used in this study were housed at either Emory University with Emory University Institutional Animal Use and Care Committee (PROTO201800112) approval, or at BIOQUAL, Inc. with BIOQUAL Institutional Animal Use and Care Committee (Study # 18-001) approval.

Note that full information on the approval of the study protocol must also be provided in the manuscript.

Flow Cytometry

Plots

Confirm that:

- [x] The axis labels state the marker and fluorochrome used (e.g. CD4-FITC).
- [x] The axis scales are clearly visible. Include numbers along axes only for bottom left plot of group (a 'group' is an analysis of identical markers).
- [x] All plots are contour plots with outliers or pseudocolor plots.
- [x] A numerical value for number of cells or percentage (with statistics) is provided.

Methodology

Sample preparation	Anticoagulated blood was obtained from NHPs at the timepoints specified. PBMC were isolated by density gradient centrifugation and stored cryopreserved in vapor phase of LN2 prior to thawing for analysis. Samples were thawed by incubating in a 37°C water bath followed by washing with warmed media before being stained with fluorochrome-labeled mAbs and tetramerized Env hooks. All reagents were titered to ensure optimal staining. B-cell hooks were combined to cover the immunogens used for each experiment. Samples were washed and resuspeneded in PBS-BSA and then sorted immediately. Detailed Standard Operating Procedure is available upon request.

Instrument	BD FACSAria II (BD Biosciences, San Jose, CA)
Software	BD FACSDiva 8.0.1
Cell population abundance	All sorting was performed using the purity setting. The sorted populations were gated as described. Target cell abundance for each NHP included in the study ranged from 0.09% to 2.7% as shown in Supplemental Figures and described in the manuscript.
Gating strategy	Geometry gates/Live/CD3(neg)/CD14(neg)/CD16(neg)/CD20(pos)/CD27(all)/IgD(neg)/Env immunogen(double positive, two fluorochromes). Form completed after initial manuscript submission. A figure exemplifying the gating strategy is included in Supplemental Material.

☒ Tick this box to confirm that a figure exemplifying the gating strategy is provided in the Supplementary Information.
